# Psychosocial Mediators between Socioeconomic Status and Dietary Restrictions among Patients Receiving Hemodialysis in Japan

**DOI:** 10.1155/2019/7647356

**Published:** 2019-04-17

**Authors:** Hidehiro Sugisawa, Toshio Shinoda, Yumiko Shimizu, Tamaki Kumagai, Hiroaki Sugisaki

**Affiliations:** ^1^Graduate School of Gerontology, J. F. Oberlin University, Machida-shi 194-0294, Japan; ^2^Faculty of Medical and Health Sciences, Tsukuba International University, Tsuchiura-city 300-0051, Japan; ^3^Faculty of Nursing, The Jikei University School of Medicine, Chofu-shi 182-8570, Japan; ^4^Graduate School of Nursing, Osaka City University, Osaka-shi 545-0051, Japan; ^5^Hachioji Azumacho Clinic, Hachioji-shi 192-0082, Japan

## Abstract

The generalizability of differences in dietary restrictions (DRs) as function of socioeconomic status (SES) and the pathways of the associations between SES and DRs remain unclear. Therefore, we aimed to explore SES differences in DRs and psychosocial mediators between SES and DRs in Japanese patients receiving hemodialysis. This study was a cross-sectional survey of 6,644 outpatients (average age = 66.5 years; 65% males) of hemodialysis facilities across Japan. DRs were assessed by self-reported and objective measures, and SES was assessed based on education and income. Three psychosocial mediators were used: self-efficacy, control expectancy, and social support. Indirect influences of SES through the mediators were evaluated with a multiple mediator model. Although higher education was significantly associated with higher self-reported DRs, higher income was significantly associated with lower self-reported DRs. Significant SES differences in objective DRs were not observed. The relationships between education and self-reported DRs and objective DRs were significantly mediated by self-efficacy and/or control expectancy. The influences of income were mediated by social support. It becomes possible to design interventions targeting modifiable psychosocial factors including self-efficacy, control expectancy, and social support in order to reduce SES inequalities in DRs.

## 1. Introduction

Socioeconomic status (SES) influences a wide range of health and health-related indicators in patients receiving dialysis. Studies using quality of life (QOL) indicators as outcome measures have shown strong relationships between higher SES and increased QOL scores [1–4]. Higher SES in patients receiving dialysis is related to mental and physical health indicators, such as lower depression [5–7], less frequent complications [5], and higher levels of activities of daily living [7] as compared to patients with a lower SES. In addition, lower SES in patients receiving dialysis is significantly related to higher mortality [8–15]. As several studies have reported that SES indicators are related to several health and health-related indicators, fluid and dietary restrictions (DRs), which are health-maintenance factors in patients receiving dialysis, may be influenced by SES. In fact, according to a recent review by Lambert and colleagues [16], SES impacted these restrictions in patients receiving dialysis. However, since there have been scant studies on SES differences in fluid/DRs, especially in oriental societies such as China and Japan, which have different cultural background from Western society, the generalizability of the previous findings remains unclear. Concurrently, the mechanisms underlying SES differences in health and fluid/DRs among patients receiving dialysis have rarely been pursued.

Psychosocial mediators that explain the influence of SES on health and health-related behaviors have been explored in diverse patient groups, such as patients with coronary heart diseases [17], diabetes [18, 19], asthma or rhinitis [20–22], myocardial infarction [23], bacteremia [24], and patients who are chronically ill [25]. In addition, several previous studies have clarified significant influences of psychosocial factors on fluid/DRs in patients receiving dialysis, including self-efficacy [26–33], social support [29, 30, 34–37], perceived barriers/benefits [38–40], and locus of control [34].

Studies of the psychosocial mediators between SES and health behaviors noted that social support [41–46], self-efficacy [42–44, 46], and perceived control [43, 46] are in inconsistently distributed across SES. Although it remains unclear whether distributions of these psychosocial factors differ by SES in patients receiving dialysis, psychosocial factors may work as mediators to explain the relationships between SES and fluid/DRs in patients receiving dialysis. Consequently, we explored not only SES differences in DRs, but also psychosocial mediators between these indicators in Japanese patients receiving hemodialysis.

We used educational attainment and income as measures of SES. While education, income, and occupational status indicators are frequently used as SES indicators [47], we excluded occupational status because most of the patients receiving hemodialysis in Japan were women or older adults, both of which were nonworkers. According to a large-scale survey conducted in 2011 involving members of the Japan Association of Kidney Disease Patients, in which a third of the patients undergoing dialysis participated, the labor force participation rate of women as well as patients over 65 years old was 16.8% and 16.0%, respectively [48].We used both education and income indicators to determine if they had distinct influences on DRs. Education appears to be a better index of psychosocial resources, such as the ability to manage social systems (e.g., navigate the healthcare system), effectively regulate health behaviors, accrue social support, and develop a sense of self-efficacy [49]. Income is related to the possession of material or immaterial resources, such as better housing, food, and resources for mastering stressful and demanding situations (e.g., seeking professional help); thus, a high income provides the opportunity for a health-promoting lifestyle [49]. If psychosocial resources such as social support and self-efficacy, which may be largely influenced by education, have an influence on DRs [50], we hypothesized that education would affect DRs through psychosocial mediators more than income would.

## 2. Materials and Methods

### 2.1. Data

We analyzed cross-sectional data collected in 2016 from all outpatients and their physicians across 118 dialysis facilities that were members of the Japan Association of Dialysis Physicians. The survey process comprised five steps. First, we requested all 923 members of the Association to participate in the survey. Second, we sent self-administrated questionnaires to the 118 dialysis facilities that agreed to participate. Third, medical staff distributed the questionnaires to all outpatients. Questionnaires included an explanation of participants' rights, privacy, and so on. Fourth, after patients completed the questionnaires, they sealed them in an envelope and handed them to their physicians (i.e., physicians could not see patients' responses). Lastly, physicians answered questionnaires regarding their patients and sent both sets of questionnaires to the survey secretariat.

Overall, we obtained questionnaires from 6,783 outpatients; however, the number of paired questionnaires—responded to by both patients and their physicians—was 6,644, which were subsequently included in our analyses.

### 2.2. Measures

#### 2.2.1. DRs

DRs were measured using both self-reported (SDRs) and objective (ODRs) measures. Shimizu and Sugisawa [51] developed an SDRs measure that included intake restrictions of three minerals: salt, phosphorus, and potassium. These three items were included in reliable and valid scales [52]. Although the original questionnaires included water restrictions, we excluded this restriction because we allowed beverages between meals. To measure restriction, we used a 4-point Likert scale:* do almost all the time* (4),* do sometimes* (3),* do a few times* (2), and* almost never do* (1). Regarding reliability, we conducted a principal component analysis for the three items using eigenvalues > 1.0 criterion. Only a single component was extracted and all principal component scores of the three items exceeded 0.75. The first component explained 68.8% of the total variance in the three items. Cronbach's alpha was .77. Regarding validity, we examined whether this measure could predict patients with the acceptable serum potassium range: lower than 6.0 mEq/l. Some studies showed that patients with 6.0 mEq/l and over had a significantly increased mortality risk [53, 54]. We also examined whether this measure could discriminate patients with serum phosphorus of 3.5–5.5 mg/dl. This range was recommended by the National Kidney foundation [55]. Maintaining serum phosphorus within this range may result in a better survival rate among patients receiving dialysis [56]. Consequently, SDRs significantly predicted an acceptable range of each medical indicator.

Levels of serum potassium and serum phosphorus were used to measure ODRs. We asked physicians to give the most recent numeric values of the two clinical indicators for each patient. As mentioned above, each criterion of acceptable DRs was lower than 6.0 mEq/l for serum potassium and 3.5–5.5 mg/dl for serum phosphorus. In prior studies, Baraz et al. [57] defined dietary compliance as serum creatinine, sodium, serum potassium, calcium, phosphate, albumin, uric acid, and blood urea nitrogen being within the acceptable ranges, and Chan et al. [58] also considered dietary compliance as both serum potassium and phosphorus being within the acceptable ranges. They defined the lowest health risk group as dietary compliant using multiple medical indicators. By following their criteria, effective DRs were judged as having both serum potassium and phosphorus levels fall within the acceptable ranges in this study.

#### 2.2.2. SES

SES was measured by educational attainment and household income. We asked participants to indicate their highest level of educational attainment: “junior high school,” “high school,” “vocational school,” “junior college,” “university,” or “graduate school.” We assigned 9, 12, 13, 14, 16, and 18 to each category to quantify their responses. These figures reflect one's number of years of education according to the Japanese educational system. Regarding income, we calculated family-size adjusted income by dividing household annual income by the square root of household size. Eight income levels from “under one million yen (US $8,900)” to “over ten million yen” (US $89,000) were used to assess household annual income. We assigned midpoints to each income category to quantify the responses.

#### 2.2.3. Possible Psychosocial Mediators for DRs

We originally created three scales to measure possible psychosocial mediators: self-efficacy, control expectancy, and social support, which were based on scales used in past research that explored psychosocial mediators between SES and dietary habits in older adults [46]. Content validity for each item was confirmed by three dialysis physicians and two patients receiving hemodialysis.

*Self-Efficacy*. Participants were asked questions about their confidence levels concerning whether they could not consume food in each of the following three scenarios: (a) when their favorite foods were right in front of them, (b) when they were very hungry, and (c) when others recommend the foods. Responses were scored using a four-point scale (1 =* not at all confident* to 4 =* very confident*). Responses were summed to provide one self-efficacy score. A principal component analysis extracted a single component by imposing an eigenvalue > 1.0 criterion, which explained 78.9% of the total variance. All principal component scores exceeded .88. Cronbach's alpha of this index was 0.88.

*Control Expectancy*. Regarding health maintenance, participants were asked to indicate the importance of (a) restricting their salt intake, (b) not eating foods high in potassium, and (c) not eating foods high in phosphorus. Responses were scored using a four-point scale (1 =* not important* to 4 =* very important*). Responses were summed to provide one control expectancy score. A principal component analysis extracted a single component by imposing an eigenvalue > 1.0 criterion, which explained 74.5% of the total variance. All principal component scores exceeded 0.80. Cronbach's alpha of this index was 0.84.

*Social Support*. Participants were asked about the level of perceived social support they received from their family/friends in each of the following three scenarios: (a) family/friends understand how hard it is to perform DRs, (b) family/friends cooperate with participants' performance, and (c) family/friends offer advice concerning DRs. Responses were scored using a four-point scale (1 =* not supportive/no support* to 4 =* very supportive*). Responses were summed to provide one social support score. A principal component analysis extracted a single component by imposing an eigenvalue > 1.0 criterion, which explained 79.7% of the total variance. All principal component scores exceeded 0.88. Cronbach's alpha for this index was 0.87.

#### 2.2.4. Control Variables

Sex (1 = male, 0 = female), age, period of dialysis, and activities of daily living were included as control variables. Period of dialysis (years) was from the initiation of dialysis to survey commencement. Activities of daily living were measured by levels of support needed to perform five activities: (1) ambulation indoors, (2) change clothes, (3) eat a meal, (4) take a bath, and (5) use a toilet. Responses ranged from “*do at his/her ease*” to “*always need support to do it as he/she cannot do it*.” Participants who chose “*do at his/her ease*” for all five activities were assigned zero points. Participants who chose “*have a little difficulty with it*” or responses with more severe difficulties for at least one activity were assigned one point. Preliminary analysis showed that neither SDRs nor ODRs were influenced by primary causes of kidney diseases and adding these variables to the analytic model decreased model fitness. Consequently, we excluded these variables from analyses.

### 2.3. Statistical Analyses

We conducted multiple mediation analyses as proposed by Preacher and Hayes [59, 60] to determine the total and specific indirect effects in a multiple factor model. Analyses were conducted with Mplus Version 8.1 software; Muthén & Muthén, Los Angeles, CA, USA [61]. In addition, we examined the null hypotheses that the indirect effects of each mediator resulting from the education and income indicators were equal [61]. We standardized all variables in the model to compare the sizes of the indirect effects produced by each mediator. We used bootstrapping (the number of bootstrap samples=5,000) to estimate the total and specific indirect effects of the mediators. We determined the point estimates and 95% confidence intervals per the null hypothesis.

We employed a full information maximum likelihood approach to handle missing data in the analysis [61]. We examined whether there were mediation effects, even if the total effects of education and income did not have significant effects on SDRs and ODRs. This approach was based on the suggestion that the significant effect of the independent variable is not always necessary for mediation to occur [59]. For example, if M_1_ acts as a mediator and a second mediator, M_2_, acts as a suppressor, the total effects of the independent variable on the dependent variable might be reduced, given the possibility that the indirect effects of M_1_ and M_2_ cancel each other out. We assessed the overall model fit using the root mean square error of approximation (RMSEA) and the comparative fit index (CFI). It is recommended that the RMSEA be below 0.05 [62], and the CFI be above 0.90 [63].

### 2.4. Ethical Considerations

This study was conducted per the guidelines of the Helsinki Declaration, and all procedures were approved by the Research Ethics Board of J. F. Oberlin University. The questionnaire, along with the letter of invitation explaining the study content, was given to each potential survey respondent. Data collection procedures assured confidentiality using self-administered, anonymous questionnaires. Responses were completely voluntary, and confidentiality was fully guaranteed. As we asked participants to return the completed questionnaires only if they agreed to participate in the survey, only respondents who wished to participate returned their questionnaires, thus implying consent.

## 3. Results

### 3.1. Descriptive Statistics

Table 1 shows participants' descriptive statistics and correlations among study variables. Sex, dialysis duration, and disabled activities of daily living were control variables. Multicollinearity was not a concern. Regarding SDRs, the path coefficients of education and income were significant after considering age, sex, activities of daily living, and other SES indicators (0.035,* P *= .013; -0.056,* P* < .001, respectively). Regarding ODRs, the path coefficients of education and income were nonsignificant after considering age, sex, activities of daily living, and other SES indicators (B = -0.020,* P *= .277; B= -0.018,* P* = .337, respectively).

### 3.2. Multiple Mediation

**Figure 1:**
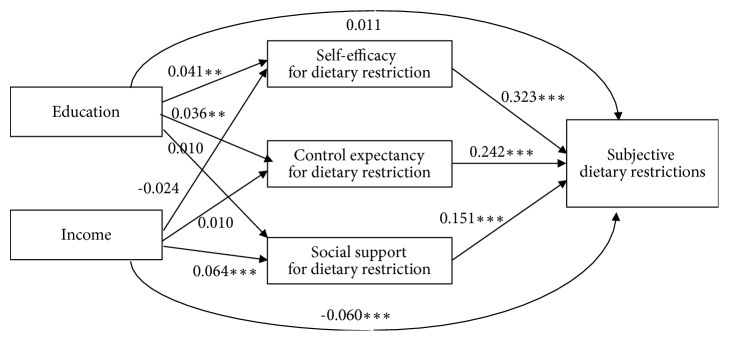
Direct and indirect effects of socioeconomic status on subjective dietary restrictions as a function of psychosocial mediators. Note  1: the effects of each variable were obtained after controlling for the influence of other variables without the variable in question. Note  2: ^*∗*^*P* < .05, ^*∗∗*^*P* < .01, and ^*∗∗∗*^*P* < .001. Note  3: total indirect influence of education on subjective dietary restrictions (SDRs) through three mediators was 0.024 (95% confidence interval: 0.009 to 0.038); each indirect influence of education on SDRs through self-efficacy, control expectancy, and social support was 0.013 (0.004 to 0.023), 0 0.009 (0.002 to 0.014), and 0.002 (-0.002 to 0.006) separately. Note  4: total indirect influence of income on SDRs through three mediators was 0.004 (95% confidence interval:-0.011 to 0.019); each indirect influence of income on SDRs through self-efficacy, control expectancy, and social support was -0.008 (-0.017 to 0.001), 0.002 (-0.005 to 0.009), and 0.010 (0.005 to 0.015) separately. Note  5: difference in indirect influences of education on SDRs through self-efficacy and control expectancy was 0.004 (-0.005 to 0.014) through self-efficacy and social support was 0.011 (0.003 to 0.021); and through control expectancy and social support was 0.007 (0.000 to 0.014). Note  6: difference in indirect influences of income on SDRs through self-efficacy and control expectancy was 0.010 (0.000 to 0.020); through self-efficacy and social support was 0.018 (0.008 to 0.027); difference in indirect influences of income on SDRs through control expectancy and social support was 0.008 (0.000 to 0.015). Note  7: root mean square error of approximation = 0.077; comparative fit index = 0.987.

Figure 1 displays the results of the multiple mediation analysis regarding SDRs. The model fit was acceptable. Most direct effects of education on self-efficacy and control expectancy as mediators of SDRs were significant. However, education had no significant direct effects on support. On the other hand, the direct effects of income only on social support were significant. Each direct effect of self-efficacy, control expectancy, and social support on SDRs was significant. Multiple mediation analyses showed that self-efficacy and control expectancy were significant mediators between education and SDRs. In addition, self-efficacy was a more significant mediator than was social support. After entering the three mediators in the analytic model, the effect of education on SDRs became smaller and nonsignificant (*P *= .374). Only social support was a significant mediator between income and SDRs; however, income did not have significant total indirect effects on SDRs. Social support was a more significant mediator than was self-efficacy. After entering the three mediators in the analytic model, the direct negative effect of income on SDRs became larger by subtracting the positive indirect effects of income (*P* < .001).

**Figure 2:**
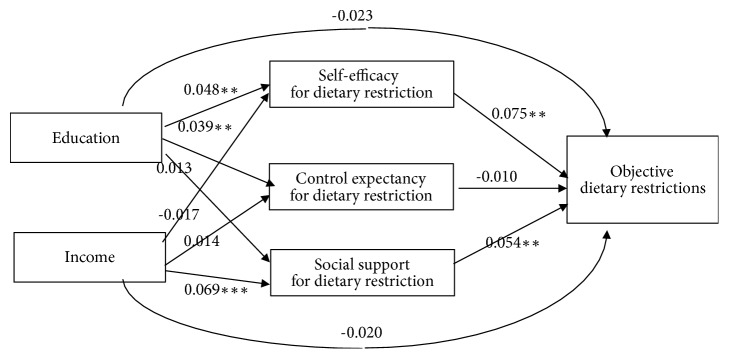
Direct and indirect effects of socioeconomic status on objective dietary restrictions as a function of psychosocial mediators. Note  1: the effects of each variable were obtained after controlling for the influence of other variables without the variable in question. Note  2: ^*∗*^*P* < .05, ^*∗∗*^*P* < .01, and ^*∗∗∗*^*P* < .001. Note  3: total indirect influence of education on objective dietary restrictions (ODRs) through three mediators was 0.004 (95% confidence interval: 0.001 to 0.008); each indirect influence of education on ODRs through self-efficacy, control expectancy, and social support was 0.004 (0.001 to 0.007), 0.000 (-0.002 to 0.001), and 0.001 (-0.001 to 0.003) separately. Note  4: total indirect influence of income on ODRs through three mediators was 0.002 (95% confidence interval:-0.001 to 0.006); each indirect influence of income on ODSs through self-efficacy, control expectancy, and social support was -0.001 (-0.004 to 0.001), 0.000 (-0.001 to 0.000), and 0.004 (0.001 to 0.007) separately. Note  5: difference in indirect influences of education on ODRs through self-efficacy and control expectancy was 0.004 (0.001 to 0.008); through self-efficacy and social support was 0.003 (0.000 to 0.006); and through control expectancy and social support was 0.001 (-0.001 to 0.004). Note  6: difference in indirect influences of income on ODRs through self-efficacy and control expectancy was 0.001 (-0.001 to 0.004); through self-efficacy and social support was 0.005 (0.002 to 0.009); and through control expectancy and social support was 0.004 (0.001 to 0.008). Note  7: root mean square error of approximation = 0.080; comparative fit index = 0.979.

Figure 2 provides the results of the multiple mediation analysis regarding ODRs. The model fit was moderately acceptable. The direct effects of both education and income on the three mediators were almost the same as the results for SDRs above. Each direct effect of self-efficacy and social support on ODRs was significant. Multiple mediation analyses showed that self-efficacy significantly mediated education and ODRs. In addition, self-efficacy was a more significant mediator than was control expectancy. After entering the three mediators in the analytic model, the direct negative effect of education on ODRs became larger by subtracting the positive indirect effects of education (*P* = .192). Regarding the effects of income, only social support was a significant mediator between income and ODRs. Social support was a more significant mediator than was self-efficacy and control expectancy. After entering the three mediators in the analytic model, the direct negative effect of income on ODRs became larger by subtracting the positive indirect effects of income (*P* = .276).

## 4. Discussion

We hypothesized that SES differences are linked with DRs in patients receiving hemodialysis. In this study, higher education significantly influenced higher SDRs after considering the influence of control variables. This result supports our hypothesis. On the other hand, income had a significant influence on SDRs after considering the influence of the control variables, displaying a significant link between higher income and lower SDRs, which contrasts our hypothesis. Some studies indicated that higher SES had significant negative influences on DRs [36, 64], while others showed significant links between higher income and higher DRs [16]. However, these authors did not explain the reasons for their results and noted the limited generalizability of their findings. Our current results are valid because of the large sample size, which was drawn from all outpatients receiving dialysis in Japan.

Why was higher income associated with lower SDRs? In this study, DR elements included phosphorus, potassium, and salts. In the general population, consumption of fruits and vegetables including consuming a lot of potassium is recommended; however, this differs for patients receiving dialysis [65]. In addition, protein-rich foods—including meat and fish—are recommended for older adults to have a healthy diet [66, 67]. Protein-rich foods contain a lot of phosphorus and potassium, although additives are increasingly being added to processed and fast foods, particularly meat, cheese, baked goods, and beverages. A review article indicated that adults with a lower SES were less likely to consume fruit and vegetables [64, 68] and being older and having a lower individual and household income predicted a decreased consumption of fruits and vegetables [69–71]. Low-income adults also consume less meat and fish than do their wealthier counterparts [71]. One explanation for these findings is that fresh fruit and vegetables are expensive [72] and income is related to the possession of material or immaterial resources, such as better housing, food, and resources for dealing with stressful and demanding situations (e.g., seeking professional help) [49]. According to these findings in the general population, results of this study indicate that it is difficult for most higher income patients receiving dialysis to change their dietary habits because they were more likely to consume potassium- and phosphorus-rich foods before they had started receiving dialysis.

One reason for the nonsignificant SES differences in ODRs might be related to taking potassium- and phosphorus-lowering medication. According to systematic review, very few studies found low education to be significantly associated with medication nonadherence [73]. Therefore, the consistency of taking this medication across SES levels may contribute to the nonsignificant differences seen in patients receiving dialysis.

We further showed that SES indicators had indirect significant influences on both SDRs and ODRs through psychosocial mediators. Our findings suggest that the mechanisms for these are distinct depending on the SES indicator, education or income. Notably, education, income, and occupational status cannot be used interchangeably in social epidemiology [47]. In fact, some studies showed that each indicator predicted distinct health indicators [47, 74, 75]. However, these past studies did not determine the relevant mechanisms behind these differences. Regarding SES differences in mediational influence, it has been suggested that education can reflect a range of noneconomic psychosocial resources effectively to regulate health behaviors, accrue social support, and develop a sense of personal control or agency [49]. Further, income is a more direct index of material resources than is education, as it reflects one's ability to afford better healthcare, nutrition, housing, and so on, thus promoting better health and recovery [49].

According to this study, education had significant influences on SDRs and ODRs through self-efficacy and/or control expectancy, and income only had significant influences on SDRs and ODRs through social support. These results support the hypothesis that education influences DRs through boosting noneconomic psychosocial recourses such as self-efficacy. However, income was also mediated by social support, although the significant mediators differed from those for education. Why did social support mediate income and both indicators of DRs? Regarding support in general, it is hypothesized that the relationship between income and social support is mediated by life events, which disrupt and impair social relationships [76]. Among people with a lower income, social network members tend to share resources (albeit few) to enhance the person's capacity to cope with the problem. Patients receiving dialysis who belong to a lower income group are likely to have experienced negative life events owing to their limited resources. Consequently, approaching their networks with multiple events may overwhelm the network's limited recourses and availability. This may be the reason for the reduced influence of social support among low-income patients receiving dialysis.

Our study may have important public health and policy implications. By understanding the mediating factors that explain the relationship between SES and DRs, it becomes possible to design interventions targeting modifiable psychosocial factors, including self-efficacy, control expectancy, and social support, in order to reduce SES inequalities in DRs. The effect of these interventions may be most beneficial for lower SES patients receiving hemodialysis.

This study had some limitations. First, although we used a large, representative sample, the income differences in SDRs contrasted our hypothesis and prior results; therefore, future studies should attempt to validate our results including examining non-Japanese patients receiving dialysis. Second, we did not determine whether participants were taking medication to decrease their phosphorus or potassium levels, which could have affected the results. Third, although we included both self-reported and objective measures to evaluate DRs, other adherence measures—such as fluid restrictions, taking medicine, and the frequency of skipping dialysis—should be examined to determine if they are associated with patients' SES. Fourth, social desirability bias (i.e., the defensive tendency to present oneself in a more favorable light) might exist in responses regarding SDRs. A study reported that there social desirability bias exists in dietary self-report questionnaires [77]. Although this study did not include social desirability in the analytic framework, there is a need to identify social desirability bias using social desirability scales.

## 5. Conclusions

The results obtained from this study cannot provide a definitive answer to whether there are SES differences in DRs in Japanese patients receiving hemodialysis. However, despite its limitations, this study provided novel findings; specifically, although patients with low levels of education who receive hemodialysis may have lower SDRs than patients with a higher level of education, it is possible that higher income patients receiving hemodialysis have lower DRs than lower income patients. Further, although the influences of SES may be mediated through self-efficacy, control expectancy, and/or social support, these mediators may differ between education and income indicators.

## Figures and Tables

**Table 1:** Descriptive statistics and correlations among study variables.

Variable	Mean	SD	1	2	3	4	5	6	7	8	9
1. Age	66.45	11.68	-								
2. Sex	0.65	0.48	-.006								
3. Duration of dialysis	10.36	9.38	−.081^*∗∗∗*^	−.098^*∗∗∗*^							
4. Disabled ADL	0.3	0.46	.286^*∗∗∗*^	-.067^*∗∗∗*^	.044^*∗∗∗*^						
5. Education	18.35	2.38	−.257^*∗∗∗*^	.157^*∗∗∗*^	.054^*∗∗∗*^	−.153^*∗∗∗*^					
6. Income	2.41	1.64	−.098^*∗∗∗*^	.041^*∗∗∗*^	.004	−.136^*∗∗∗*^	.310^*∗∗∗*^				
7. SE	7.90	1.89	.126^*∗∗∗*^	−.027^*∗*^	.027^*∗*^	−.009	.001	-.019			
8. CE	9.67	1.58	.000	−.081^*∗∗∗*^	−.041^*∗∗∗*^	−.036^*∗∗*^	.024	.026^*∗*^	.342^*∗∗∗*^		
9. SS	8.95	1.92	.236^*∗∗∗*^	.031^*∗*^	−.080^*∗∗∗*^	.054^*∗∗∗*^	−.030^*∗*^	.046^*∗∗∗*^	.261^*∗∗∗*^	.258^*∗∗∗*^	
10. Self-reported DRs	8.81	1.56	.167^*∗∗∗*^	−.078^*∗∗∗*^	−.063^*∗∗∗*^	.061^*∗∗∗*^	−.041^*∗∗∗*^	−.063^*∗∗∗*^	.455^*∗∗∗*^	.395^*∗∗∗*^	.316^*∗∗∗*^
11. Objective DRs	0.63	-	-	-	-	-	-	-	-	-	-

Note 1: SD: standard deviation, ADL: activities of daily living, SE: self-efficacy for dietary restrictions, CE: control expectancy for dietary restrictions, SS: social support for dietary restrictions, and DRs: dietary restrictions.

Note 2: means and correlations were calculated using the full-information maximum likelihood method.

Note 3: means and standard deviations represent values before the variables were standardized.

Note 4: objective dietary restrictions represent a dependent and categorical variable; we did not calculate its correlations with the independent variables.

Note 5: ^*∗*^*P* < .05, ^*∗∗*^*P* < .01, and ^*∗∗∗*^*P* < .001.

## Data Availability

The data are available from the corresponding author upon reasonable request.
